# Physical Determinants of Interval Sprint Times in Youth Soccer Players

**DOI:** 10.2478/hukin-2014-0013

**Published:** 2014-04-09

**Authors:** William E. Amonette, Denham Brown, Terry L. Dupler, Junhai Xu, James J. Tufano, John K. De Witt

**Affiliations:** 1 Human Performance Laboratory, Department of Clinical, Health, and Applied Sciences, University of Houston – Clear Lake, Houston, TX.; 2 Houston Dynamo Academy, Houston, TX.

**Keywords:** Speed, Acceleration, Talent Identification, Soccer Screening, Soccer

## Abstract

Relationships between sprinting speed, body mass, and vertical jump kinetics were assessed in 243 male soccer athletes ranging from 10–19 years. Participants ran a maximal 36.6 meter sprint; times at 9.1 (10 y) and 36.6 m (40 y) were determined using an electronic timing system. Body mass was measured by means of an electronic scale and body composition using a 3-site skinfold measurement completed by a skilled technician. Countermovement vertical jumps were performed on a force platform - from this test peak force was measured and peak power and vertical jump height were calculated. It was determined that age (r=−0.59; p<0.01), body mass (r=−0.52; p<0.01), lean mass (r=−0.61; p<0.01), vertical jump height (r=−0.67; p<0.01), peak power (r=−0.64; p<0.01), and peak force (r=−0.56; p<0.01) were correlated with time at 9.1 meters. Time-to-complete a 36.6 meter sprint was correlated with age (r=−0.71; p<0.01), body mass (r=−0.67; p<0.01), lean mass (r=−0.76; p<0.01), vertical jump height (r=−0.75; p<0.01), peak power (r=−0.78; p<0.01), and peak force (r=−0.69; p<0.01). These data indicate that soccer coaches desiring to improve speed in their athletes should devote substantive time to fitness programs that increase lean body mass and vertical force as well as power generating capabilities of their athletes. Additionally, vertical jump testing, with or without a force platform, may be a useful tool to screen soccer athletes for speed potential.

## Introduction

Speed and speed endurance are essential physical characteristics for successful match-play (McIntyre, 2005; Bangsbo et al., 2007) in the sport of soccer. Over the course of a game, players must accelerate and decelerate rapidly, jump, bound, and leap repeatedly. Moreover, players must maintain the ability to accelerate, decelerate, and change direction for the entire duration of match play, which may last up to 90 minutes or more while maintaining soccer-specific skills (Reilly, 1997; Shephard, 1999). All of these motor skills and movements require athletes to generate large quantities of force over short periods of time (Thorlund et al., 2009). Therefore, speed and power are important components of soccer-specific fitness training (Jakobsen et al., 2012; Amonette et al., 2012). To determine the physical characteristics associated with power and speed in youth soccer players, there are many available field and laboratory tests (Unnithan et al., 2012). Analyzing the data from these tests can aid coaches or athletes in developing soccer-specific fitness programs, promoting anthropometric changes, and identifying players with athletic skill sets that increase their probability of sport success (Lango-Penas et al., 2011; Mirkov et al., 2010). Running speed is a function of stride length and the stride rate (Chapman and Caldwell, 1983; Krysztop and Mero, 2013; Miller et al., 2012). Research indicates the magnitude of the ground reaction force (Weyand et al., 2000) and the rate of force development during the stance phase of sprinting are the primary determinants of stride length and of top-speed capabilities (Weyand et al., 2000). Therefore, with appropriate technical training for sprinting, soccer players with greater force and power generating capabilities (i.e. greater muscle mass) may possess the ability to run faster than athletes with lower force and power generating capabilities (Alexander, 1989).

The ability to sprint quickly is also related to body mass. A massive athlete must generate more force to accelerate their mass at a given rate compared to a less massive athlete (Newton’s 2^nd^ law). Therefore, the accumulation of too much mass may inhibit one’s ability to accelerate and achieve high running velocities. The vertical jump is a field-based test that evaluates an athlete’s ability to generate ground reaction force and since an athlete must vertically propel their body mass it is a good assessment of force generating capabilities. Since the muscle groups and actions used in the vertical jump are similar to sprinting movements, athletes who can generate greater forces may be capable of reaching greater sprint speeds (Young et al., 1995). The vertical jump test can be used as a field based assessment when evaluating jump height alone, or it can be instrumented to evaluate the kinetics of the jump including force and power production (Amonette et al., 2012). Each may be important in evaluating speed potential. The purpose of this study was to determine the relationship between interval running speed, player anthropometrics, and vertical jump kinetics in youth soccer players. Second, we determined the strength of association of field-derived anthropometic and performance variables alone to sprint time at 9.1 and 36.6 m using a multiple regression model. We hypothesized that sprint time at 9.1 and 36.6 m would be significantly correlated with age, percent fat, body mass, lean mass, vertical jump height, power, and force. Moreover, we hypothesized that time to 9.1 and 36.6 m could be accurately predicted using a liner combination of field-derived variables: age, body mass, lean mass, and vertical jump height.

## Material and Methods

### Participants

Anthropmetric and performance data were collected on 243 male athlete participants (14.4 ± 1.9 y; 58.6 ± 12.9 kg; 14.6 ± 0.1% fat) at a youth soccer camp. The participants were competing for selection into an elite youth soccer academy. The sample included players who ranged in age from 10 – 19 y (Table 1) and comprised all participants at the camp; therefore, the total number of individuals within age categories was unequal. Because the players were not rostered on the academy team prior to data collection, no data were available concerning the player position or their level of soccer experience. Prior to participation, each player and their parental guardian completed a medical history screening form and signed informed consent agreeing to participate. The study procedures, protocols and informed consent forms were approved by the Committee for the Protection of Human Subjects (CPHS) at the sponsoring university in accordance with the Declaration of Helsinki.

### Measures and Procedures

All subjects completed the following tests in consecutive order: body composition determination, body mass determination, vertical jump, and 36.6 meter sprint (40 yards). The tests were selected because they are routinely used to assess human performance and may be indicative of soccer-specific fitness. The 40 yard sprint (36.6 meters) was used, as opposed to 40 meters, because the youth academy from which the data were collected historically assessed speed from this distance. Therefore, it was necessary to standardize the distance for retrospective comparison of academy athletes.

Body composition data were obtained by a skilled technician using Lange skinfold calipers (Beta Technology Inc., Cambridge, MA). Skinfold (SF) measurements were determined from the following sites on the right side of the body: pectoralis, abdomen, and thigh. The pectoralis skinfold measurement was completed diagonally, mid-way between the acromioclavicular joint line and the nipple. The abdomen skinfold was measured vertically, two inches lateral to the umbilicus. The thigh skinfold was determined with a vertical pinch mid-way between the superior pole of the patella and greater tronchanter of the femur. Body density was determined using a generalized equation for males; subsequently, %fat was computed using Siri’s equation (Thompson, 2010).

Vertical jump was completed by each participant while standing on a force platform (Accupower, AMTI, Watertown, MA). After a soccer-specific warm-up, participants were provided with verbal instruction on how to properly complete the test. Then, they were given 2–3 practice vertical jumps of increasing intensity. When ready, the subjects completed a single maximal vertical jump while holding 0.40 kg wooden dowel across their posterior deltoids to eliminate any upper limb contribution to jump height (Amonette et al., 2012). Prior to performance of the jump, the force platform was zeroed and the subject was weighed. Then, the athletes rapidly bent their knees and hips to a self-selected depth and jumped as high as possible landing on the force platform. The test was repeated if there was a problem with data collection or an obvious mechanical error by the participating athlete.

Vertical jump height, peak force, and peak power were determined using a commercial software program (Dartpower 1.0, Athletic Republic, Park City, UT). The program used the measured ground reaction force to compute the velocity of the center of mass. Vertical jump height was computed using the take-off velocity of the center of mass and a projectile motion equation. Power was computed as the instantaneous velocity of the center of mass multiplied by the measured ground reaction force. Peak force (PF) was determined as the maximum measured force in the vertical axis during the acceleration phase of the jump. Likewise, peak power (PP) was the maximum instantaneous power during the acceleration of the jump.

Speed was measured during a 36.6 m linear sprint (Vescovi et al., 2011). During the sprint, split-times were determined at 9.1 m and total time was measured at 36.6 m using electronic timing gates (Newtest *Inc*., Turku, Finland). After a warm-up, participants performed two timed sprints from which the fastest time was selected and used for further analysis. When ready, the participants stood on a marked line in a staggered two point stance with a self-selected foot forward. The time was triggered at the athlete’s initial movement and was stopped when the athlete’s center of mass passed through the timing gates at 9.1 and 36.6 m.

### Statistical Analysis

Data were initially entered into Microsoft Excel and checked for errors and anomalies. Then, all statistical analyses were performed using SigmaPlot 12.0 (Systat Inc, San Jose, CA). Pearson’s correlations were computed between time at 9.1 and 36.6 meters and the following variables: age (y), percent fat (%), body mass (BM; kg), lean mass (LM; kg), vertical jump height (VJ; cm), peak power (PP; W), and peak force (N). To assess the second study hypothesis, a multiple regression equation was derived to predict time at 9.1 and 36.6 m using the field expedient measurements (i.e. Age, BM, LM, and VJ) via backwards elimination. A critical variable inflation factor (VIF) of ≥5.0 was defined *a priori* to indicate co-linearity and subsequent elimination of variables. A significance level of *p*≤0.05 was used to determine statistical significance. The following qualitative scale was used to assess strength of association: strongly correlated (*r*>0.70), moderately correlated (*r*=0.30–0.69), and weakly correlated (*r*<0.29) (Riffenburgh, 2012).

## Results

Means and standard deviations for each tested variable by age group are provided in Table 1. No statistical comparisons were completed between age-groups since this was not directly tied to the study hypotheses. Time at 9.1 m was moderately correlated with age (*r*=−0.59; *p*<0.01), BM (*r*=−0.52; *p*<0.01), LM (*r*=−0.61; *p*<0.01), VJ (*r*=−0.67; *p*<0.01), peak power (*r*=−0.64; *p*<0.01), and peak force (*r*=−0.56; *p*<0.01). Percent fat was weakly, but significantly correlated with time at 9.1 m (*r*=0.25; *p*<0.01). Similarly, time at 36.6 m was strongly correlated with age (*r*=−0.71; *p*<0.01), LM (r=−0.76; *p*<0.01), VJ (*r*=−0.75; *p*<0.01), and peak power (*r*=−0.78; *p*<0.01). Body mass (*r*=−0.67; *p* < 0.01) and peak force (*r*=−0.69; *p*<0.01) were moderately correlated with 36.6 m time. Percent fat was weakly, but significantly correlated with time at 36.6 m (*r*=0.17; *p*<0.01).

**Figure 1.**
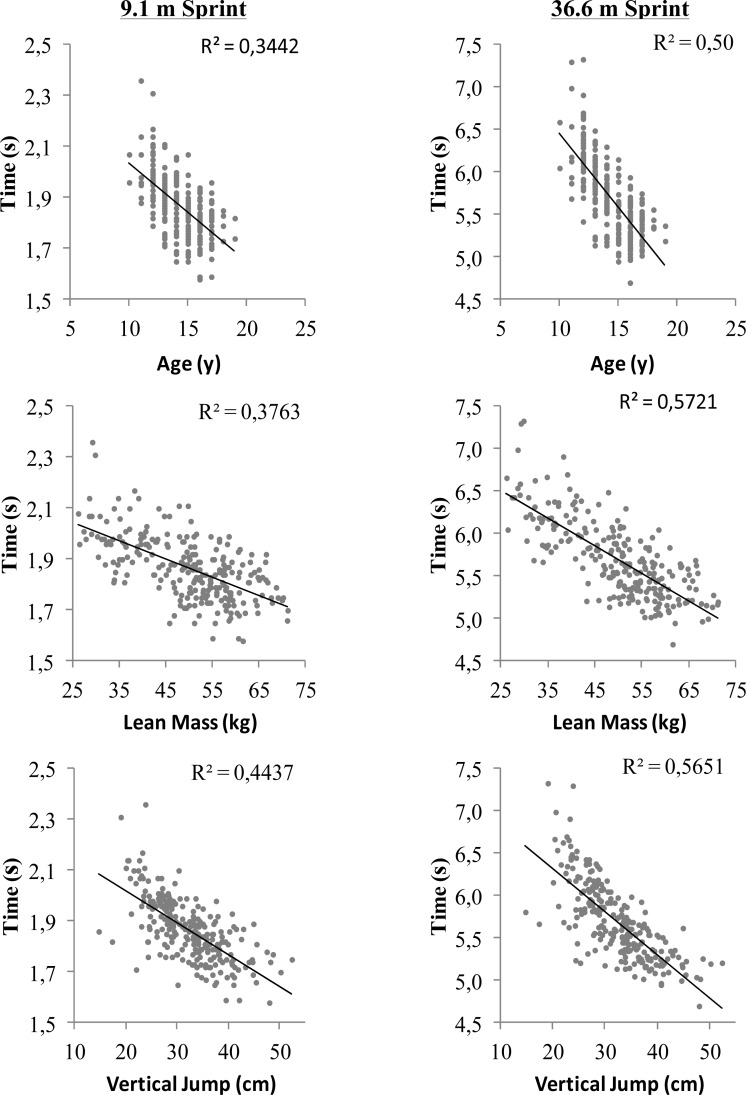
Relationship between time at 9.1 and 36.6 m to age (y), lean mass (kg), and vertical jump (cm) in the 243 male soccer players.

Final regression equations, derived via backward elimination, can be seen in Table 2. The table includes the variable coefficiants and standard error of the measurements (s) for the two equations. Body mass was eliminated from the 9.1 and 36.6 m multiple regression equation due to colinearity with lean mass (VIF > 5.0). No other variables were co-linear. The final multiple regression equation for 9.1 m time (r=0.71) included age (*p*=0.01), lean mass (*p*<0.001), and VJ (*p*<0.001); the equation explained 50% of the variance in time to 9.1 m. The multiple regression equation for 36.6 m (r=0.84) included age (*p* < 0.001), lean mass (*p* < 0.001), and VJ (*p* < 0.001). The equation explained 71% of the variance in time to 36.6 m. A scatter plot depicting the relationship between age, lean mass, and VJ to time at 9.1 and 36.6 m can be seen in Figure 1.

## Discussion

The purposes of this investigation were to determine the relationship between athlete anthropometrics, vertical jump kinetics and speed at 9.1 and 36.6 m in young soccer players. We also sought to determine the relationship between the field-derived variables (age, body mass, lean mass, and VJ height) to speed at 9.1 and 36.6 m. These data indicate a clear and strong relationship between both laboratory and field derived variables to time at 9.1 and 36.6 m. The relationship is more apparent at the 36.6 compared to 9.1 m.

Several investigators have studied the relationship between vertical jump kinetics, body mass and speed in soccer, American football, softball, and rugby players. Most of these studies sampled collegiate to professional level players and demonstrated strong relationships between body mass and speed (Baker and Newton, 2008; Nimphius et al., 2010; Sawyer et al., 2002; Shalfawi et al., 2011). In general, as body mass increases athletes tend to be slower (Nimphius et al., 2010; Sawyer et al., 2002). This is not surprising since force production to impart acceleration to an object is proportional to the mass of the object (Newton’s 2^nd^ Law). In contrast to previous findings, we found that participant athletes in our sample who were larger tended to be faster than smaller athletes. There are two possible explanations for this finding. Since previous investigators used older, more mature athletes the differences in size were likely determined primarily by excess fat weight. In our sample, the larger subjects possessed more lean mass and thus had greater force generating potential than the smaller subjects. Second, it is reasonable that the larger subjects were older and thus we demonstrated an age-affect and not a lean body mass affect. However, the multiple-regression modeling indicated that age and lean body mass were independent predictors of speed in youth soccer players; therefore, it is possible, if not likely, that both age and lean body mass affected speed differently.

Similar to the findings of previous investigators, sprint times were moderately to highly correlated with force and power production in the vertical jump (Vescovi et al., 2011). Previous research has shown that top-speed in sprinting is related to the magnitude of the ground reaction force during the propulsion phase of sprinting (Weyand et al., 2000). Thus, the greater the force that an individual is able to impart on the ground with each step, the greater distance covered during each stride.

We used a force platform and a simple vertical jump test to determine the peak force and peak power capabilities of our athletes. Our findings demonstrate that both peak force and peak power during a vertical jump are highly correlated with speed. Thus, athletes who are able to generate greater force and power during a vertical jump probably generate a greater ground reaction force during sprinting (Weyand et al., 2000). Although we used a force platform to measure force and calculate power in the vertical jump, field expedient equations have been developed to estimate power production using field-derived data (Amonette et al., 2012). Such equations may be useful in estimating peak power capabilities of athletes when a force platform is not available for testing and may provide useful information for soccer coaches identifying athletes with greater speed potential.

The field-derived equations for estimating speed were accurate at both 9.1 m (SEM = 0.08 s) and 36.6 m (SEM = 0.24 s) in this investigation. This finding suggests that speed can be accurately estimated using only the age, lean mass, and vertical jump height of the subject. The reported equations could be used in testing situations when running a 36.6 m sprint is impractical or when time is limited for testing. Future research is needed to cross-validate these equations in different populations.

We found that age, body mass, lean mass, vertical jump height, power, and force were all better predictors of speed at 36.6 m compared to 9.1 m (i.e. higher correlations). There are several possible explanations for this finding. First, the mechanics of top-speed sprinting, and associated force vectors are more similar to the vertical jump than the acceleration phase of sprinting. More specifically, the acceleration phase of sprinting involves a greater proportion of horizontal ground reaction forces compared to vertical. Previous research has demonstrated that broad jump may be a better predictor of shorter distance speeds than vertical jump (Robbins, 2012). This finding is likely because the forces in the broad jump are more similar to sprint acceleration than the vertical jump. Future research may investigate the relationship between broad jump and shorter distance sprint times in youth soccer players.

A second possible reason for this finding is that the athletes in this investigation were not trained in sprint starting mechanics. The only instructions provided to the subjects were to begin with their toe and a marked line and to run as fast as possible for the entire 36.6 m. Since sprint starting mechanics are highly technical, it could be that the young athletes had little instruction in the movements and struggled with the technical nature of the start. If this reasoning is valid, it may be advantageous to include resistance training exercises and running drills that incorporate rapid acceleration to overcome static inertia in youth soccer players.

A third explanation is that sprinting from a stopped position is dissimilar to most soccer-specific movements. In the game of soccer, a player rarely accelerates from a complete stopped position to full speed. Instead, most plays necessitate that an athlete is running at a slower velocity and then speeds up or changes directions and sprints to full speed. A better method of assessing interval sprint speed may be to assess times using a “flying” 36.6 m sprint. Such a test would eliminate the mechanics of sprinting and more completely assess the construct of speed.

## Conclusions

In conclusion, we found that sprint speed is moderately to strongly correlated with age, body mass, lean mass, vertical jump height, peak power, and peak force. The relationship of each of these variables to speed seems to be stronger with longer running distances in youth soccer players. Quantification of age, body mass, lean mass, and vertical jump height and associated kinetics may be useful in identifying athletes with the potential for greater speed. In contrast to previous findings, these data suggest that body mass, and more specifically larger amounts of lean mass, may improve speed in youth soccer players. To improve speed, coaches may choose to implement fitness training regimens that increase lean body mass and improve vertical ground reaction force capabilities.

## Figures and Tables

**Table 1:** Cross sectional data by age group reported as mean ± standard deviation.

	9.1 m (s)	36.6 m (s)	Lean Mass (kg)	Vertical Jump (cm)	Power (W)	Force (N)
<12y (n=10)	2.03 ± 0.14	6.33 ± 0.52	31.7 ± 4.2	26.3 ± 4.4	1473.6 ± 279.7	515.5 ± 108.7
12y (n=36)	2.00 ± 0.10	6.27 ± 0.36	37.8 ± 6.7	26.0 ± 3.5;	1766.3 ± 460.7	563.9 ± 188.1
13y (n=54)	1.91 ± 0.11	5.91 ± 0.32	42.6 ± 7.0	28.1 ± 4.3	2126.6 ± 524.9	687.3 ± 192.3
14y (n=39)	1.86 ± 0.11	5.68 ± 0.29	50.5 ± 6.4	32.3 ± 5.4	2689.1 ± 568.1	798.6 ± 165.6
15y (n=36)	1.81 ± 0.10	5.47 ± 0.30	54.9 ± 5.5	34.6 ± 4.3	3083.1 ± 516.0	881.7 ± 221.2;
16y (n=44)	1.80 ± 0.08	5.39 ± 0.25	58.0 ± 5.6	37.0 ± 6.1	3478.8 ± 706.4	968.7 ± 208.4
17y (n=17)	1.79 ± 0.09	5.36 ± 0.21	59.3 ± 6.5	36.9 ± 6.7	3325.2 ± 605.1	883.1 ± 212.5

**Table 2:** Multiple regression equations derived using backwards elimination using age (y), lean mass (LM; kg), and vertical jump height (VJ; cm)

**Equation**	**SEM**	**R**
9.1 m (s) = −0.0109_age_ − 0.00235_LM_ − 0.00815_VJ_ + 2.404	0.08	0.71
36.6 m (s) = −0.0474_age_ − 0.0141_LM_ − 0.0285_VJ_ + 8.006	0.24	0.84

## References

[b1-jhk-40-113] Alexander MJ (1989). The relationship between muscle strength and sprint kinematics in elite sprinters. Can J Sport Sci.

[b2-jhk-40-113] Amonette WE, Brown LE, De Witt JK, Dupler TL, Tran TT, Tufano JJ, Spiering BA (2012). Peak vertical jump power estimations in youths and young adults. J Strength Cond Res.

[b3-jhk-40-113] Baker DG, Newton RU (2008). Comparison of lower body strength, power, acceleration, speed, agility, and sprint momentum to describe and compare playing rank among professional rugby league players. J Strength Cond Res.

[b4-jhk-40-113] Bangsbo J, Iaia FM, Krustrup P (2007). Metabolic response and fatigue in soccer. Int J Sports Physiol Perform.

[b5-jhk-40-113] Chapman AE, Caldwell GE (1983). Kinetic limitations of maximal sprinting speed. J Biomech.

[b6-jhk-40-113] Jakobsen MD, Sundstrup E, Randers MB, Kjaer M, Andersen LL, Krustrup P, Aagaard P (2012). The effect of strength training, recreational soccer and running exercise on stretch-shortening cycle muscle performance during countermovement jumping. Hum Mov Sci.

[b7-jhk-40-113] Krzysztof M, Mero A (2013). A kinematics analysis of three best 100 m performances ever. J Hum Kinet.

[b8-jhk-40-113] Lago-Penas C, Casais L, Dellal A, Rey E, Dominguez E (2011). Anthropometric and physiological characteristics of young soccer players according to their playing positions: relevance for competition success. J Strength Cond Res.

[b9-jhk-40-113] McIntyre MC (2005). A comparison of the physiological profiles of elite Gaelic footballers, hurlers, and soccer players. Br J Sports Med.

[b10-jhk-40-113] Mendez-Villanueva A, Buchheit M, Kuitunen S, Douglas A, Peltola E, Bourdon P (2011). Age-related differences in acceleration, maximum running speed, and repeated-sprint performance in young soccer players. J Sports Sci.

[b11-jhk-40-113] Miller RH, Umberger BR, Caldwell GE (2012). Sensitivity of maximum sprinting speed to characteristic parameters of the muscle force-velocity relationship. J Biomech.

[b12-jhk-40-113] Mirkov DM, Kukolj M, Ugarkovic D, Koprivica VJ, Jaric S (2010). Development of anthropometric and physical performance profiles of young elite male soccer players: a longitudinal study. J Strength Cond Res.

[b13-jhk-40-113] Nimphius S, McGuigan MR, Newton RU (2010). Relationship between strength, power, speed, and change of direction performance of female softball players. J Strength Cond Res.

[b14-jhk-40-113] Reilly T (1997). Energetics of high-intensity exercise (soccer) with particular reference to fatigue. J Sports Sci.

[b15-jhk-40-113] Riffenburgh RH (2012). Statistics in Medicine.

[b16-jhk-40-113] Robbins DW (2012). Relationships between National Football League combine performance measures. J Strength Cond Res.

[b17-jhk-40-113] Sawyer DT, Ostarello JZ, Suess EA, Dempsey M (2002). Relationship between football playing ability and selected performance measures. J Strength Cond Res.

[b18-jhk-40-113] Shalfawi SA, Sabbah A, Kailani G, Tonnessen E, Enoksen E (2011). The relationship between running speed and measures of vertical jump in professional basketball players: a field-test approach. J Strength Cond Res.

[b19-jhk-40-113] Shephard RJ (1999). Biology and medicine of soccer: an update. J Sports Sci.

[b20-jhk-40-113] Thompson W (1992). ACSM’s Guidelines for Exercise Testing and Prescription.

[b21-jhk-40-113] Thorlund JB, Aagaard P, Madsen K (2009). Rapid muscle force capacity changes after soccer match play. Int J Sports Med.

[b22-jhk-40-113] Unnithan V, White J, Georgiou A, Iga J, Drust B (2012). Talent identification in youth soccer. J Sports Sci.

[b23-jhk-40-113] Vescovi JD, Rupf R, Brown TD, Marques MC (2011). Physical performance characteristics of high-level female soccer players 12–21 years of age. Scand J Med Sci Sports.

[b24-jhk-40-113] Weyand PG, Sternlight DB, Bellizzi MJ, Wright S (2011). Faster top running speeds are achieved with greater ground forces not more rapid leg movements. J Appl Physiol.

[b25-jhk-40-113] Young W, McLean B, Ardagna J (1995). Relationship between strength qualities and sprinting performance. J Sports Med Phys Fitness.

